# Analytical Solution of Multicompartment Solute Kinetics for Hemodialysis

**DOI:** 10.1155/2013/654726

**Published:** 2013-11-06

**Authors:** Przemysław Korohoda, Daniel Schneditz

**Affiliations:** ^1^AGH University of Science and Technology, Faculty of Computer Science, Electronics and Telecommunications, Department of Electronics, al. A. Mickiewicza 30, 30-059 Krakow, Poland; ^2^Institute of Physiology, Center for Physiological Medicine, Medical University of Graz, Harrachgasse 21/5, 8010 Graz, Austria

## Abstract

*Objective.* To provide an exact solution for variable-volume multicompartment kinetic models with linear volume change, and to apply this solution to a 4-compartment diffusion-adjusted regional blood flow model for both urea and creatinine kinetics in hemodialysis. *Methods.* A matrix-based approach applicable to linear models encompassing any number of compartments is presented. The procedure requires the inversion of a square matrix and the computation of its eigenvalues **λ**, assuming they are all distinct. This novel approach bypasses the evaluation of the definite integral to solve the inhomogeneous ordinary differential equation. *Results.* For urea two out of four eigenvalues describing the changes of concentrations in time are about 10^5^ times larger than the other eigenvalues indicating that the 4-compartment model essentially reduces to the 2-compartment regional blood flow model. In case of creatinine, however, the distribution of eigenvalues is more balanced (a factor of 10^2^ between the largest and the smallest eigenvalue) indicating that all four compartments contribute to creatinine kinetics in hemodialysis. *Interpretation.* Apart from providing an exact analytic solution for practical applications such as the identification of relevant model and treatment parameters, the matrix-based approach reveals characteristic details on model symmetry and complexity for different solutes.

## 1. Introduction

Compartment models are popular in pharmacokinetics and, as a special application, in hemodialysis, where such models serve to quantify treatment dose [1–3]. Most of that kinetic analysis has been done for urea usually described by 2-compartment models. However, unlike most pharmacokinetic models, the volume of compartments cannot be assumed as constant because of ultrafiltration of excess volume within and accumulation of volume between hemodialysis treatments. The effects on solute concentrations and solute balance caused by volume changes are not negligible. Still, the problem can be expressed as 2-dimensional, inhomogeneous ordinary differential equations (ODE) [4], and the closed form solution to this problem is known. Furthermore, for the variable-volume 2-compartment model urea concentrations have been presented as explicit functions of time and model parameters so that the concentrations in the two compartments at any time can be computed in a single step [5, 6]. That approach was based on the variation of the constants method.

**Figure 1:**
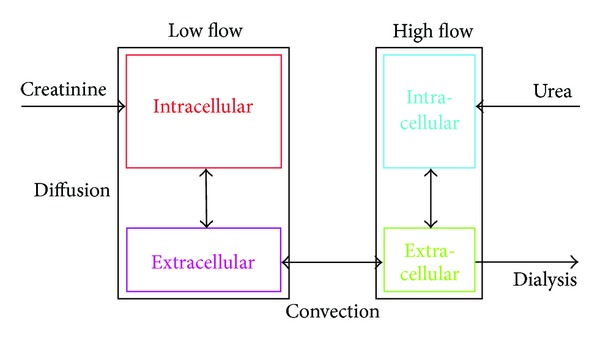
Schematic representation of the four-compartment DA-RBF model [12]; note that the blood flow is not exposed; for detailed description see formulas in the Appendix.

While urea, a solute of little toxicity, is a useful marker of uremia, solutes with limited transfer between compartments such as phosphate, creatinine, *β*_2_-microglobulin, or glucose are of greater clinical interest [7–11]. Recently, one of us presented a variable-volume 4-compartment model to describe the kinetics of both urea and creatinine using physiological principles of solute transport and only one parameter (membrane permeability) to distinguish between the two solutes [12] (Figure 1). Again, the model can be presented as a 4-dimensional, inhomogeneous ODE, for which the general solution involves integration and which is not defined in the closed form. Thus, the concentrations of the solutes in the four compartments are not obtained without extensive computations.

It is the purpose of this paper to provide an exact analytical solution for multicompartment kinetic problems, assuming linear changes in volume, and to apply this solution to the recently described 4-compartment model for both urea and creatinine kinetics in hemodialysis.

## 2. Methods

The *N*-compartment model with time dependent volume values, *v*_*n*_(*t*), and concentration of the solute, *c*_*n*_(*t*), can be described by the following set of differential equations:

(1) ddt(vn(t)cn(t))=kn1c1(t)+kn2c2(t)+⋯+knNcN(t)+gn,                     n=1,2,…,N,

where coefficients *k*_*nm*_ describe the solute exchange or removal in terms of solute clearance, typically in mL/min, and *g*_*n*_ models solute mass input to the *n*th compartment in terms of generation rate, typically in mg/min. After substitution using

(2) $$\begin{array}{c} b_{nm}=\{\begin{array}{cc} k_{nm} & \text{for}\;m\neq n\\ k_{nn}-\frac{d}{dt}v_{n}\left(t\right) & \text{for}\;m=n, \end{array} \end{array}$$

we obtain

(3) vn(t)ddtcn(t)=bn1c1(t)+⋯+bnncn(t)+⋯+bnNcN(t)+gn,                       n=1,2,…,N.

The set of equations from (3) can be rewritten in matrix form

(4) $$\begin{array}{c} v\left(t\right)\circ \frac{d}{dt}c\left(t\right)=Bc\left(t\right)+g, \end{array}$$

where capital symbols are used for matrices, small symbols in bold represent vectors, and ∘ denotes the Hadamard product [13].

Assume that the volume changes are described by linear functions of time:

(5) $$\begin{array}{c} v\left(t\right)=\left(V_{0}+Qt\right)r:\sum_{n=1}^{N}r_{n}=1; \end{array}$$

here *V*_0_ is the total volume of all compartments at time *t* = 0, *Q* refers to the negative ultrafiltration rate, *Q* < 0, during hemodialysis (HD), or to the positive fluid accumulation rate, *Q* > 0, during the interdialytic interval (ID), and *r*_*n*_ refers to the fractional volume of each compartment.

Next, let us normalize the volume to the initial value [5]:

(6) $$\begin{array}{c} w\left(t\right)=\frac{V_{0}+Qt}{V_{0}}. \end{array}$$

Notice that *w*(*t*) is a linear function of *t*. Then, after having replaced elements of matrix **B** and vector **g** with the following values

(7) $$\begin{array}{c} a_{nm}=\frac{b_{nm}}{r_{n}Q},\;\;b_{n}=\frac{g_{n}}{r_{n}Q}, \end{array}$$

(4) may be rewritten as

(8) $$\begin{array}{c} \frac{V_{0}}{Q}w\left(t\right)\frac{d}{dt}c\left(t\right)=Ac\left(t\right)+b. \end{array}$$

The analytical expressions for the concentrations defined by (8), for *n* = 1,2,…, *N*, assuming that matrix **A** has *N* distinct eigenvalues, are in the form

(9) cn(t)=xn1wλ1(t)+xn2wλ2(t)+⋯+xnNwλN(t)+dn,

where *λ*_*n*_ are the eigenvalues of matrix **A**. The matrix equation equivalent to (9) is given as

(10) $$\begin{array}{c} c\left(t\right)=Xw^{\lambda }\left(t\right)+d, \end{array}$$

where *w* ^***λ***^(*t*) is a column vector consisting of elements *w* ^*λn*^(*t*).

### 2.1. The Proof

**Theorem 1**

To prove that for a system described by (8), the solution is provided by (9) or (10).

**Lemma 2**

Consider the general matrix form of the vector differential equation to be solved as

(11) $$\begin{array}{c} \left(p+qt\right)\frac{d}{dt}c\left(t\right)=Ac\left(t\right)+b\text{,}\;p+qt\neq 0. \end{array}$$

For simplification assume that all *N* eigenvalues of matrix **A** are distinct.

One will show that, under assumption that **b** belongs to the image of a transform defined by matrix **A** which is true for the considered model, the solution of (11) is in the form of

(12) c(t)=(p+qtp)(1/q)A(c0−d)+d,where  d  satisfies  condition:b=−Ad.

**Sublemma**

First, let one show that

(13) $$\begin{array}{c} Au^{A}=u^{A}A. \end{array}$$

Recall that the Hermite polynomial for the function of a matrix *f*(**A**) [14], also known as Lagrange-Sylvester formula for the case when all *N* eigenvalues are distinct, is given as

(14) $$\begin{array}{c} f\left(A\right)=\sum_{n=1}^{N}f\left(\lambda_{n}\right)\prod_{\begin{array}{c} k=1\\ k\neq n \end{array}}^{N}\frac{\left(A-\lambda_{k}I\right)}{\left(\lambda_{n}-\lambda_{k}\right)}, \end{array}$$

where *λ*_*n*_ is the *n*th eigenvalue of matrix **A**, so that *f*(*λ*_*n*_) is the scalar function of a scalar variable. Assuming that *u* is scalar, and taking *f*(**A**) = *u* ^**A**^, one obtains that *u* ^**A**^ is a sum of scalars multiplied by integer powers of **A**, showing that (13) must be true. This observation ends the proof of the sublemma.

With (13) we obtain that the following identity, which results from substituting the right-hand term of (12) into right-hand side of (11), is also true:

(15) $$\begin{array}{c} Ac\left(t\right)+b=A{\left(\frac{p+qt}{p}\right)}^{(1/q)A}c_{0}+{\left(\frac{p+qt}{p}\right)}^{(1/q)A}b. \end{array}$$

Now, let us find, using (12), the expression for the derivative of **c**(*t*):

(16) $$\begin{array}{c} \frac{d}{dt}c\left(t\right)=\frac{1}{p+qt}A{\left(\frac{p+qt}{p}\right)}^{(1/q)A}\left(c_{0}-d\right). \end{array}$$

To explain the result in (16) let us consider the scalar *u* being

(17) $$\begin{array}{c} u=u\left(t\right)={\left(\frac{p+qt}{p}\right)}^{1/q}. \end{array}$$

The derivative of the scalar function *u* ^*λ*^(*t*) results in another function *f*(*λ*):

(18) f(λ)=ddtuλ(t)=λu(t)uλ(t)ddtu(t),where  for  p+qt≠0:ddtu(t)=ddt(p+qtp)1/q   =1qpp+qt(p+qtp)1/qddt(p+qtp)   =1qpp+qtu(t)qp=1p+qtu(t).

Utilizing again (14), we obtain that the derivative of *u* ^**A**^(*t*) must be

(19) $$\begin{array}{c} f\left(A\right)=\frac{d}{dt}u^{A}\left(t\right)=\frac{1}{u\left(t\right)}Au^{A}\left(t\right)\frac{d}{dt}u\left(t\right). \end{array}$$

Substituting *u*(*t*) from (17) to (19) and using the result from (18) we get (16).

Comparing the right-hand sides of (15) and (16), we obtain that **c**(*t*) given by (12) satisfies (11). If we substitute *t* = 0 in (12) we obtain that also the initial condition is satisfied.

Expansion from (14) used for *f*(**A**) = *u* ^**A**^ leads also to the identity

(20) $$\begin{array}{c} u^{A}x_{n}=x_{n}u^{\lambda_{n}}, \end{array}$$

where **x**_*n*_ is the *n*th eigenvector of matrix **A** corresponding to the eigenvalue *λ*_*n*_. Equation (20) implies that

(21) $$\begin{array}{c} u^{A}\sum_{n=1}^{N}y_{n}s_{n}=\sum_{n=1}^{N}y_{n}s_{n}u^{\lambda_{n}}, \end{array}$$

where **y**_*n*_ is the normalized *n*th eigenvector and *s*_*n*_ is the scaling coefficient relating **y**_*n*_ to **x**_*n*_. As it is always possible to represent the *N*-dimensional vector in the relevant basis, we can write the following:

(22) $$\begin{array}{c} c_{0}-d=\sum_{n=1}^{N}y_{n}s_{n}. \end{array}$$

Assuming (17) and comparing (21), (22), and (12) we obtain that the analytical solution of (11) is in the form of

(23) c(t)=d+∑n=1Nynsnuλn(t)  =  y1s1uλ1(t)+y2s2uλ2(t)+⋯+yNsNuλN(t)+d.

In the discussed dialysis models, where *p* + *qt* = (*V*_0_/*Q*)*w*(*t*) = (*V*_0_ + *Qt*)/*Q*, we take *q* = 1 and *p* = *V*_0_/*Q*, and in such case the general solution (11) can be reduced to the form

(24) $$\begin{array}{c} c\left(t\right)=w^{A}\left(t\right)\left(c_{0}-d\right)+d. \end{array}$$

Thus, in (23) *u*(*t*) should be replaced with *w*(*t*), which ends the proof of the theorem.

### 2.2. The Computational Recipe

The practical computational algorithm to compute the coefficients in (9) or in (10) is presented below.

**Step 1**

Solve the linear equation and find **d**:

(25) $$\begin{array}{c} Ad=-b\Rightarrow d=A^{-1}\left(-b\right). \end{array}$$

**Step 2**

Find the eigenvalues *λ*_*n*_ of matrix **A** and check if they are distinct. If they are, proceed with Step 3; if not, which should be an extremely rare case, return to the model parameters and introduce small changes in their values; then start from formulating the set of equations.

**Step 3**

Find the corresponding eigenvectors **y**_*n*_, and form the following square matrix **Y**:

(26) $$\begin{array}{c} Y=\left[\begin{array}{cccc} y_{1} & y_{2} & \cdots & y_{N} \end{array}\right]. \end{array}$$

**Step 4**

Solve another linear equation and find the set of *N* scaling coefficients *s*_*n*_ matching the initial conditions **c**_0_ = **c** (*t* = 0):

(27) $$\begin{array}{c} Ys=\left(c_{0}-d\right)\;\Rightarrow \;s=Y^{-1}\left(c_{0}-d\right). \end{array}$$

**Step 5**

Compute scaled eigenvectors **x**_*n*_ to obtain coefficients *x*_*nm*_:

(28) $$\begin{array}{c} x_{n}=s_{n}y_{n}\;\Rightarrow \;X=\left[\begin{array}{cccc} x_{1} & x_{2} & \cdots & x_{N} \end{array}\right]. \end{array}$$

After having computed the concentrations for all compartments, in hemodialysis modeling it is quite useful to compute the so-called equilibrated solute concentration, proportionally averaged for all compartments:

(29) $$\begin{array}{c} c_{\text{eq}}\left(t\right)=\sum_{n=1}^{N}r_{n}c_{n}\left(t\right), \end{array}$$

which represents the overall state of the patient in terms of solute concentration and which is directly related to treatment dose [15–17]. Another concentration of interest is that of the solute in the arterial plasma representing the concentration accessible to direct experimental measurements. See ((A.12)) in the Appendix for the relevant formula. 

The schematic representation of the DA-RBF model, for which the described algorithm was developed, is presented in Figure 1. 

The basic computations were performed for two sets of reference model parameters, for urea and creatinine, contained in Table 1.

To verify whether the assumptions required in the designed algorithm are reasonable and to check whether the observed properties are more general, a comparative study was performed with model parameters randomly varied around the reference values within the physiologically justified range. Pseudo random values were generated for assumed intervals according to uniform distribution. Thus, for *f*_*p*_ and *f*_pw_ a 2% radius around the central value was assumed, for *f*_ew_ 5%, for *f*_QH_ and *E*_*x*_ 10%, and 50% for *Q*_*c*_, *Q*_*f*_, *Q*_*x*_, *V*_*x*_, *H*, *f*_VH_, *f*_ecv_, *V*_*t*_, *C*_0_, and *G*_24_. Two conditions were added to prevent extremely irrelevant cases: (a) 0.01*Q*_*x*_ < *V*_*t*_, (b) *V*_uf_ < 0.125*V*_*t*_. For example, *Q*_*c*_ ranged from 2.9 L/min to 8.7 L/min. In such a way two sets of 100,000 models were obtained, separately for urea and creatinine. 

## 3. Results

For the purpose of this study it was assumed that the whole treatment cycle was balanced with regard to volume; that is, the total water volume uptake between treatments was the same as the volume removed during ultrafiltration [12].

For the 4-compartment DA-RBF model the set of explicit expressions for matrix **B** is provided in Appendix. Then, the matrix describing the set of differential equations, (8), for the HD and ID periods, respectively, consists only of the constant values and takes the following form:

(30) $$\begin{array}{c} A_{\text{HD}}=\left[\begin{array}{cccc} a_{11} & a_{12} & a_{13} & 0\\ a_{21} & -a_{21} & 0 & 0\\ a_{31} & 0 & a_{33} & a_{12}\\ 0 & 0 & a_{21} & -a_{21} \end{array}\right],\\ A_{\text{ID}}=\left[\begin{array}{cccc} a_{11} & a_{12} & a_{13} & 0\\ a_{21} & -a_{21}-1 & 0 & 0\\ a_{31} & 0 & a_{33} & a_{12}\\ 0 & 0 & a_{21} & -a_{21}-1 \end{array}\right]. \end{array}$$

The resulting values of relevant *a*_11_ to *a*_44_ elements as functions of the model parameters, taken from Table 1, for both hemodialysis and interdialytic intervals and for urea as well as for creatinine are given in Table 2.

The corresponding eigenvalues are summarized in Table 3. Notice that all eigenvalues are negative for the interdialytic phase and positive for the hemodialysis phase. Also notice the range in eigenvalues for different solutes (up to the range of 1 × 10^6^ for urea) and phases of a complete treatment cycle. In the considered cases all the eigenvalues proved to be distinct, which resulted in four different eigenvectors. 

The above observations were confirmed for all comparative, randomly generated models. In particular, in all modeled cases matrix **A** proved to be diagonalizable, and the eigenvalues and eigenvectors were always real (not complex).

**Figure 2:**
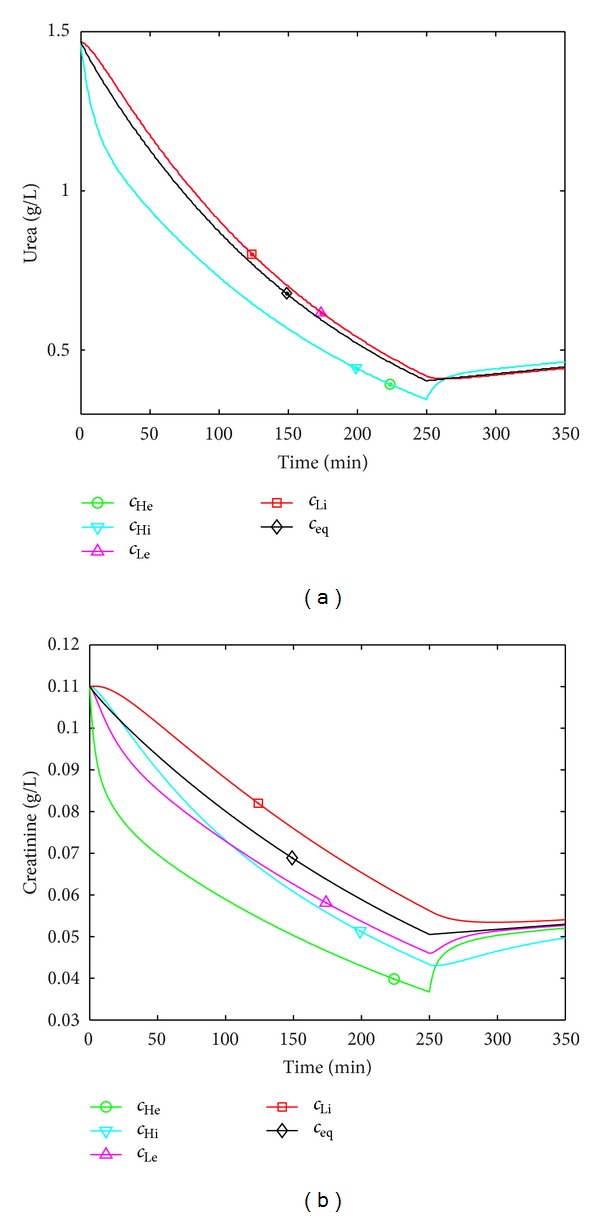
Time course of solute concentrations (urea (a); creatinine (b)) in the four compartments during hemodialysis and during a 100 min postdialytic phase using model parameters from Table 1. Notice that extra- and intracellular concentrations are superimposed in case of urea (a) and separated in case of creatinine (b). *c*_Li_: low-flow intracellular; *c*_Le_: low-flow extracellular; *c*_Hi_: high-flow intracellular; *c*_He_: high-flow extracellular; *c*_eq_: equilibrated over throughout all compartments.

Since for *t* > 0 relative volume *w* is *w* < 1 during hemodialysis and *w* > 1 during the interdialytic period, the term *w* ^*λ*^ in (9) is always 0 < *w* ^*λ*^ < 1 during both hemodialysis and interdialytic periods because of positive or negative eigenvalues, respectively. However, with very large values of **λ** such as with urea where *λ*_3_ and *λ*_4_ are in the range of 1 × 10^6^, *w* ^*λ*^ ≈ 0. This indicates that the split in intra- and extracellular spaces is ineffective in the case of urea (i.e., the intercompartment clearance is very high) and that urea follows 2-compartment kinetics. Notice the close correspondence of intra- and extracellular urea concentrations (Figure 2(a)). In case of creatinine, however, the ratio of the largest to the smallest eigenvalue is *≈*100 during hemodialysis. This indicates that all compartments contribute to overall creatinine kinetics during hemodialysis and somewhat less during the interdialytic phase. Notice the separation of concentrations in all four compartments throughout hemodialysis (Figure 2(b)).

For the 2-compartment model the matrix **A** and the eigenvalues **λ** for the 2-compartment model have been published previously [5]. A comparison of eigenvalues from the 4- to those determined from the 2-compartment model shows close correspondence for urea. Eigenvalues are in the same order of magnitude such as *λ*_1_ for the intradialytic phase with values of 15.4 and 12.3, respectively (Table 3). The small difference originates from differences in parameters assumed in published 2- and 4-compartment models which were obtained in different studies [5, 12]. When the fraction of extracellular volume was assumed as close to 1, equivalent to eliminating the effect of intracellular sequestration in the 4-compartment model, the eigenvalues for the urea model remained essentially unchanged.

**Figure 3:**
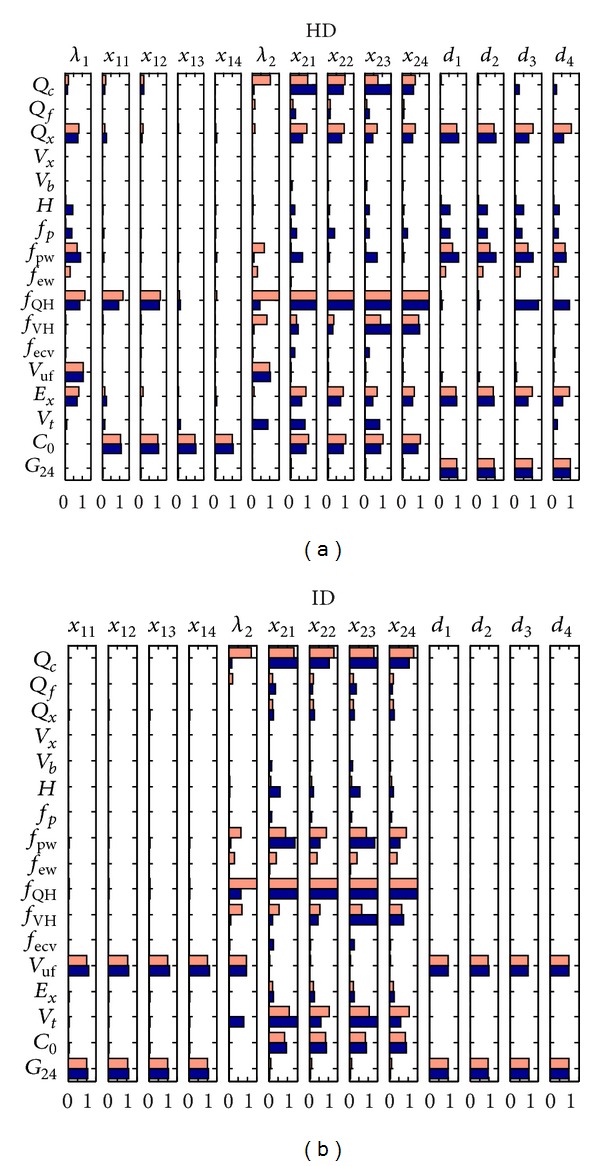
Sensitivity of computed coefficients *x*, *d*, and *λ*, with regard to model parameters for urea (upper bars in light red) and creatinine (lower bars in dark blue) models. Normalized sensitivities are indicated on the horizontal axis.

Figure 3 presents the sensitivities, expressed as absolute values, of two crucial eigenvalues and related coefficients that may have impact on the shapes of the concentration profiles when the values presented in Table 1 are varied by a small fraction. Notice that changes in *E*_*x*_ directly related to dialysis also affect the runs during the ID phase because the concentrations at the end of the HD phase serve as starting points for the ID stage.

The equilibrated concentration (29) representing the weighted average of compartmental concentrations shows the dominating impact of the first two eigenvalues.  Table 4 presents the relative error of *c*_eq_(*t*) computed at the beginning of HD for the set of 100,000 simulations using only two eigenvalues and corresponding coefficients compared to that computed with the complete set of eigenvalues. Notice the negative error with omission of positive values.

## 4. Discussion

In this paper a general analytical solution for a particular class of variable-volume multicompartment solute kinetic models is presented. The solution is based on a matrix approach applicable to linear models encompassing any number of compartments assuming that all eigenvalues of the matrix are distinct. The presented solution is based on a finite volume change typical for hemodialysis. The solution in absence of a volume change requires a different approach the discussion of which is beyond the scope of this paper. The detailed procedure is also provided.

One purpose of mathematical modeling is to characterize the system by its structure such as the number of compartments and their interaction and by its parameters such as the distribution volumes and rate constants [18]. Many parameters are usually inaccessible to direct measurement and must be obtained by parameter identification, that is, by fitting an appropriate model output to observable experimental data. This procedure involves recurrent numerical solution of the ODE for different sets of parameter values until a chosen set of parameters provides the best fit. While parameter identification in more complex models is a problem of its own [19], the numerical solution of a given ODE for each parameter set to be evaluated in the process of parameter identification makes this task very laborious and time consuming, even for powerful personal computers. It therefore pays to replace numerical for exact analytical procedures wherever possible.

The solution developed in this study was applied to the variable-volume 2-compartment model for urea kinetics as well as to the variable-volume 4-compartment model for urea and creatinine kinetics presented elsewhere [5, 12]. The latter has also been used to describe the kinetics of *β*_2_-microglobulin [10].

Apart from its mathematical use, the qualitative examination of matrices and eigenvalues provides a good means to compare models and to judge the effective number of compartments. Based on the eigenvalues ranging from 12.3 to 1.1 × 10^6^ for the hemodialysis interval, the 4-compartment urea kinetic model essentially is a 2-compartment urea kinetic model. In the case of creatinine, however, the eigenvalues range from 7.5 to 725.9 for the hemodialysis interval and are much closer to each other so that the 4-compartment structure is justified (Figure 2). The models with varied parameters indicated comparable properties.

Results presented in Table 4 confirm that computations can considerably be simplified for experimental applications. Reduction of parameters to compute *c*_eq_, important from the diagnostic and planning point of view, leading to just two eigenvalues with corresponding coefficients, should provide acceptable accuracy.

The sensitivity study shows that in clinical practice, where collection of the complete set of personalized model parameters is not always possible, the most attention should be paid to an accurate estimation of *f*_QH_, *Q*_*c*_, *V*_*t*_, *V*_uf_, *Q*_*x*_, *E*_*x*_, *C*_*o*_, *G*, *f*_pw_, and  *f*_VH_. However, limited sensitivity to other parameters indicates that uncertainty in such parameters does not considerably change the modeling and treatment outcome.

Analytical solutions for the variable-volume 2-compartment model have been presented before. In the approach presented by Grandi et al. the system of first-order linear differential equations comparable to that given in (4) was transformed into a type of Euler-Cauchy second-order differential equation and solved analytically [6]. An expansion of this method to more dimensions has not been provided.

A matrix-based strategy to solve the variable-volume 2-compartment model has been presented by one of the authors [5]. This approach has been applied to two physiologically distinct interpretations of urea kinetics, either assuming diffusion-limited transfer between intra- and extracellular compartments [20] comparable to that of Grandi et al. [6] or flow-limited transfer between poorly and highly perfused organ systems [5, 21].

In these variable-volume models, fluid is proportionally removed from both compartments. While this assumption is compatible with the flow-limited interpretation, the assumption is at odds with clinical and physiological understanding in the diffusion-limited interpretation of 2-compartment urea kinetics. In the classic diffusion-limited model the two compartments refer to intra- and extracellular volumes, and since excess fluid is best removed under close to isotonic conditions during hemodialysis [22–24] and accumulated under isotonic conditions by matched ingestion of salt and water between treatments [25], fluid is more or less exclusively removed from and added to the extracellular compartment, leaving the volume of the intracellular compartment unchanged. The classic variable-volume 2-compartment urea kinetic model therefore assumes a constant intracellular volume during hemodialysis and between dialysis treatments [1]. To account for this effect Smye and Will allocated 95% of the volume changes to the extracellular compartment and provided an approximated solution for this model [26].

For solutes actually sequestered in the intracellular space because of limited membrane permeability, transport is both diffusion- and flow-limited. The combination of both diffusion- and flow-limited transport characteristics therefore leads to a 4-compartment model presented earlier [12]. The equations can be analyzed using the matrix method.

The matrix-based strategy presented in this paper is akin to that presented in [5] albeit distinct from the previous method regarding an important technical point. As in the previous approach, the solution requires the computation of the eigenvalues of **A**. For the 2-compartment model this refers to solving a quadratic equation, for the 3-compartment model to solving a cubic equation according to Cardano [27, 28], and for the 4-compartment model to solving a quartic equation according to Tignol and Ferrari [28, 29]. For models involving more than four compartments the eigenvalues have to be found by numeric approximation using dedicated software such as Matlab (The MathWorks Inc., Natick, Massachusetts, USA). As in the previous approach, the solution requires the computation of **A** ^−1^, the inverse of matrix **A**. The manual inversion of a 2 by 2 matrix is easily done for the 2-compartment model but prohibitively tedious for matrices of higher dimensions. Dedicated software such as Matlab or internet resources can be used for that problem. The computational tool, to compute the coefficients and concentrations, developed in Java, may be accessed at [30]. An important difference to the previous approach, however, is the absence of computing the definite integral (the integration of the inverse transformation matrix from the start of the observation phase to the point of interest, equation C15 in [5]) over the time course. If the integral is not solved analytically as in [5] the integration has to be carried out numerically, defeating the purpose of an exact solution and consuming considerable computational time.

## 5. Conclusions

In conclusion, a closed-form solution for the variable-volume *N*-compartment model is presented. The solution can be applied to the variable-volume 4-compartment diffusion-adjusted regional blood flow model. Even though the complexity of models and mathematics considerably increases in the transition from 2- to 4-compartment models, the availability of an exact solution should help in practical applications such as the identification of relevant model and treatment parameters in hemodialysis.

## Figures and Tables

**Table 1:** Initial conditions, times, and model parameters of the DA-RBF model adapted from [12].

*C*_c *t*0_	0.11	g/L	Creatinine concentration at *t* = 0
*C*_u *t*0_	1.47	g/L	Urea concentration at *t* = 0
*t*_0_	0	min	Treatment start
*t*_*d*_	250	min	Treatment duration
*t*_di_	48∗60	min	Duration of interval between treatments
*V*	28.6	L	Urea distribution volume after ultrafiltration
*G*_24c_	0.00983	mol/24 h	Creatinine generation rate
*G*_24u_	0.31	mol/24 h	Urea generation rate
*Q*_*c*_	5.80	L/min	Cardiac output
*Q*_*f*_	1.00	L/min	Fistula flow
*Q*_*x*_	0.327	L/min	Extracorporeal blood flow
*V*_*x*_	0.25	L	Extracorporeal blood volume
*V*_*b*_	5.90	L	Blood volume
*H*	0.37		Hematocrit
*f*_*p*_	0.96		Fraction of packed cell volume
*f*_pw_	0.93		Plasma water fraction
*f*_ew_	0.72		Erythrocyte water fraction
*f*_QH_	0.85		Fraction of high flow blood flow
*f*_VH_	0.20		Fraction of high flow volume
*f*_ecv_	0.33		Fraction of extracellular volume
*V*_uf_	3.46	L	Ultrafiltration volume
*E*_*x*_	0.80		Dialyzer extraction
*k*_sc_	0.022	min^−1^	Specific rate constant for creatinine
*k*_su_	158.000	min^−1^	Specific rate constant for urea

**Table 2:** Elements *a*_nm_ of **A**.

	2HDu^†^	2IDu^†^	4HDu	4IDu	4HDc	4IDc
*a*_11_	292.9	−3986.7	6.636*E* + 05	−6.980*E* + 06	637.92	−5502.54
*a*_12_	−233.2	3985.7	−6.629*E* + 05	6.974*E* + 06	−92.30	971.02
*a*_13_			−550.84	6007.30	−417.30	4530.52
*a*_14_			0	0	0	0
*a*_21_	−57.72	997.09	−3.265*E* + 05	3.435*E* + 06	−44.46	478.26
*a*_22_	63.97	−998.09	3.265*E* + 05	−3.435*E* + 06	44.46	−479.26
*a*_23_			0	0	0	0
*a*_24_			0	0	0	0
*a*_31_			−135.38	1501.83	−102.04	1132.63
*a*_32_			0	0	0	0
*a*_33_			6.630*E* + 05	−6.975*E* + 06	200.62	−2104.65
*a*_34_			−6.629*E* + 05	6.974*E* + 06	−92.30	971.02
*a*_41_			0	0	0	0
*a*_42_			0	0	0	0
*a*_43_			−3.265*E* + 05	3.435*E* + 06	−44.46	478.26
*a*_44_			3.265*E* + 05	−3.435*E* + 06	44.46	−479.26

*a*_nm_: *n*th row and *m*th column element of **A**; 2: 2-compartment model; 4: 4-compartment model; HD: hemodialysis interval; ID: interdialytic interval; u: urea; c: creatinine; ^†^values for 2-compartment model taken from [5].

**Table 3:** Eigenvalues **λ** of **A**.

	2HDu^†^	2IDu^†^	4HDu	4IDu	4HDc	4IDc	4HDu*	4IDu*
*λ*_1_	15.4	−1	12.3	−1	7.5	−1	12.3	−1
*λ*_2_	341.4	−4983.83	278.6	−2477.8	38.4	−404.7	278.8	−2479.0
*λ*_3_			9.89*E* + 05	−1.0409*E* + 07	155.6	−1450.3	326828.7	−3.4382*E* + 06
*λ*_4_			9.90*E* + 05	−1.0414*E* + 07	725.9	−6709.7	326828.9	−3.4383*E* + 06

*λ*: eigenvalue; 2: 2-compartment model; 4: 4-compartment model; HD: hemodialysis interval; ID: interdialytic interval; u: urea; c: creatinine; ^†^values for 2-compartment model taken from [5]; *4-compartment model evaluated for *f*_ecv_ = 0.999.

**Table 4:** Relative error for *c*_eq_, in %, for the creatinine model during the HD phase, when *c*_eq_ was computed using only two eigenvalues and corresponding coefficients using parameters from 100,000 simulations.

*t*, min	0	10	20	30	40	50
Mean	−0.66	−0.29	−0.16	−0.09	−0.05	−0.03
SD	0.34	0.20	0.13	0.08	0.05	0.03
Minimum	−2.34	−1.50	−0.99	−0.65	−0.43	−0.28

*c*_eq_: equilibrated concentration; HD: hemodialysis interval; ID: interdialytic interval. For urea all values were below 1.0*e* − 6, and they remained small during ID for both solutes.

## Appendix

The complete set of formulas for the DA-RBF model is provided below. See Table 1 for description of the parameters.

To simplify the notation, auxiliary parameters are introduced for the volume ratios:

(A.1) r1=fVHfecv,r2=fVH(1−fecv),r3=(1−fVH)fecv,r4=(1−fVH)(1−fecv).

The volume flows during hemodialysis (HD) and interdialytic (ID) intervals are defined as

(A.2) Quf=Vuftd,Qin=Vuftdi−td,then  Q={−Quffor  HD,Qinfor  ID.

The regional blood flows, according to [12], are

(A.3) $$\begin{array}{c} Q_{s}=Q_{c}-Q_{f},\\ Q_{H}=Q_{s}f_{\text{QH}},\\ Q_{L}=Q_{s}\left(1-f_{\text{QH}}\right). \end{array}$$

Characteristic times for extracorporeal, regional, and transmembrane flows according to [12] are

(A.4) $$\begin{array}{c} \tau_{x}=\frac{V_{x}}{Q_{x}},\\ \tau_{H}=\frac{V_{b}f_{\text{VH}}}{Q_{H}},\\ \tau_{L}=\frac{V_{b}\left(1-f_{VH}\right)}{Q_{L}},\\ \kappa =\frac{k_{s}\left(\left(f_{\text{ew}}/f_{\text{pw}}\right)\left(H/\left(1-H\right)\right)+1\right)}{f_{\text{ew}}}, \end{array}$$

and the diffusion volume flow fractions for regional and extracorporeal blood flows are given as

(A.5) $$\begin{array}{c} f_{jx}=\left(1-Hf_{p}\right)f_{\text{pw}}+Hf_{p}\left(1-\exp\left(-\kappa \tau_{x}\right)\right)f_{\text{ew}},\\ f_{jH}=\left(1-H\right)f_{\text{pw}}+H\left(1-\exp\left(-\kappa \tau_{H}\right)\right)f_{\text{ew}},\\ f_{jL}=\left(1-H\right)f_{\text{pw}}+H\left(1-\exp\left(-\kappa \tau_{L}\right)\right)f_{\text{ew}},\\ f_{jv}=f_{\text{QH}}f_{jH}+\left(1-f_{\text{QH}}\right)f_{jL},\\ f_{ja}=\frac{f_{jv}Q_{s}+f_{jx}Q_{x}}{Q_{s}+Q_{x}},\\ f_{jf}=\frac{f_{jx}Q_{x}}{Q_{f}}+f_{ja}\left(1-\frac{Q_{x}}{Q_{f}}\right). \end{array}$$

The diffusion volume and convective flow rates for HD and ID intervals are computed as

(A.6) $$\begin{array}{c} F_{H}=Q_{H}f_{jH},\\ F_{L}=Q_{L}f_{jL},\\ F_{c}=Q_{c}f_{ja},\\ F_{f}=Q_{f}f_{jf},\\ F_{\text{uf}H}=Q_{\text{uf}}f_{\text{VH}},\\ F_{\text{uf}L}=Q_{\text{uf}}\left(1-f_{\text{VH}}\right),\\ F_{\text{ufHi}}=Q_{\text{uf}}f_{\text{VH}}\left(1-f_{\text{ecv}}\right),\\ F_{\text{ufLi}}=Q_{\text{uf}}\left(1-f_{\text{VH}}\right)\left(1-f_{\text{ecv}}\right),\\ F_{\text{in}H}=-Q_{\text{in}}f_{\text{VH}},\\ F_{\text{in}L}=-Q_{\text{in}}\left(1-f_{\text{VH}}\right),\\ F_{\text{inHe}}=-Q_{\text{in}}f_{\text{VH}}f_{\text{ecv}},\\ F_{\text{inLe}}=-Q_{\text{in}}\left(1-f_{\text{VH}}\right)f_{\text{ecv}},\\ F_{\text{inHi}}=-Q_{\text{in}}f_{\text{VH}}\left(1-f_{\text{ecv}}\right),\\ F_{\text{inLi}}=-Q_{\text{in}}\left(1-f_{\text{VH}}\right)\left(1-f_{\text{ecv}}\right) \end{array}$$

and intercompartment and dialyzer clearances as

(A.7) $$\begin{array}{c} K_{c}=k_{s}\left(1-f_{\text{ecv}}\right)V,\\ K_{H}=K_{c}f_{\text{VH}},\\ K_{L}=K_{c}\left(1-f_{\text{VH}}\right),\\ K_{d}=Q_{x}f_{jx}E_{x}+Q_{\text{uf}}\left(1-E_{x}\right), \end{array}$$

where *V* is the total volume at the end of HD (Table 1).

Then, the elements of **B** (4) for the hemodialysis interval are given as

(A.8) b11=−FH−KH+FH(FH+FufH)Fc−Ff+Kd,b12=KH,b13=FH(FL+FufL)Fc−Ff+Kd,b21=KH−FufHi,b22=−KH+FufHi,b31=FL(FH+FufH)Fc−Ff+Kd,b33=−FL−KL+FL(FL+FufL)Fc−Ff+Kd,b34=KL,b43=KL−FufLi,b44=−KL+FufLi,

while for the ID interval they are given as

(A.9) b11=−FH−KH+FHFHFc−Ff+FinHe,b12=KH,b13=FHFLFc−Ff,b21=KH,b22=−KH+FinHi,b31=FLFHFc−Ff,b33=−FL−KL+FLFLFc−Ff+FinLe,b34=KL,b43=KL,b44=−KL+FinLi.

The generation rate, expressed in mg/mL, in the four compartments is defined as

(A.10) g1=0,g2=Gurea,g3=0,g4=0

for urea and as

(A.11) g1=0,g2=0,g3=0,g4=Gcreatinine

for creatinine, respectively.

Note that in the above formulas indices 1 through 4 refer to extracellular high-flow (He), intracellular high-flow (Hi), extracellular low-flow (Le), and intracellular low-flow (Li) compartments, respectively (also see Figure 1). Therefore, the accessible arterial plasma concentration for the intradialytic period is given as

(A.12) cp,art(t)=cHe(t)(FH+FufH)+cLe(t)(FL+FufL)Fc−Ff+Kd.

For the interdialytic phase: *K*_*d*_ = *F*_uf*H*_ = *F*_uf*L*_ = 0.
